# Identification and validation of a novel signature based on cell-cell communication in head and neck squamous cell carcinoma by integrated analysis of single-cell transcriptome and bulk RNA-sequencing

**DOI:** 10.3389/fonc.2023.1136729

**Published:** 2023-05-05

**Authors:** Jian Wang, Hong-Cun Sun, Cheng Cao, Jian-Dao Hu, Jing Qian, Tao Jiang, Wen-Bo Jiang, Shao Zhou, Xiao-Wen Qiu, Hong-Li Wang

**Affiliations:** Department of Otorhinolaryngology Head and Neck Surgery, The Affiliated People’s Hospital of Ningbo University, Ningbo, Zhejiang, China

**Keywords:** cell-cell communication, HNSCC, single-cell transcriptome analysis, CD8+ T cell, Tumor-associated macrophage

## Abstract

**Background:**

The heterogeneous crosstalk between tumor cells and other cells in their microenvironment means a notable difference in clinical outcomes of head and neck squamous cell carcinoma (HNSCC). CD8+ T cells and macrophages are effector factors of the immune system, which have direct killing and phagocytosis effects on tumor cells. How the evolution of their role in the tumor microenvironment influences patients clinically remains a mystery. This study aims to investigate the complex communication networks in the HNSCC tumor immune microenvironment, elucidate the interactions between immune cells and tumors, and establish prognostic risk model.

**Methods:**

20 HNSCC samples single-cell rna sequencing (scRNA-seq) data and bulk rna-seq data were derived from public databases. The “cellchat” R package was used to identify cell-to-cell communication networks and prognostic related genes, and then cell-cell communication (ccc) molecular subtypes were constructed by unsupervised clustering. Kaplan-Meier(K-M) survival analysis, clinical characteristics analysis, immune microenvironment analysis, immune cell infiltration analysis and CD8+T cell differentiation correlation analysis were performed. Finally, the ccc gene signature including APP, ALCAM, IL6, IL10 and CD6 was constructed based on univariate Cox analysis and multivariate Cox regression. Kaplan-Meier analysis and time-dependent receiver operating characteristic (ROC) analysis were used to evaluate the model in the train group and the validation group, respectively.

**Results:**

With CD8+T cells from naive to exhaustion state, significantly decreased expression of protective factor (CD6 gene) is associated with poorer prognosis in patients with HNSCC. The role of macrophages in the tumor microenvironment has been identified as tumor-associated macrophage (TAM), which can promote tumor proliferation and help tumor cells provide more nutrients and channels to facilitate tumor cell invasion and metastasis. In addition, based on the strength of all ccc in the tumor microenvironment, we identified five prognostic ccc gene signatures (cccgs), which were identified as independent prognostic factors by univariate and multivariate analysis. The predictive power of cccgs was well demonstrated in different clinical groups in train and test cohorts.

**Conclusion:**

Our study highlights the propensity for crosstalk between tumors and other cells and developed a novel signature on the basis of a strong association gene for cell communication that has a powerful ability to predict prognosis and immunotherapy response in patients with HNSCC. This may provide some guidance for developing diagnostic biomarkers for risk stratification and therapeutic targets for new therapeutic strategies.

## Graphical Abstract

In this study, widely heterogeneous ligand-receptor interactions between tumor cells and other cells were identified using publicly available single-cell transcriptome data and bulk rna-sequencing. By constructing molecular subtypes of cell-cell communication (CCC) genes, the differences of CD8+ T state and other immune cells in inflammatory infiltration, immunotherapy and clinical characteristics were investigated. With CD8+T cells from naive to exhaustion state, significantly decreased expression of CD6 gene is associated with poorer prognosis in patients with HNSCC.The role of macrophages in the tumor microenvironment has been identified as tumor-associated macrophage, which can promote tumor proliferation and facilitate tumor cell invasion and metastasis. In addition, We identified five prognostic CCC related signature, which was identified as independent prognostic factor. Our study highlights the propensity for crosstalk between tumors and other cells, and we have developed a novel signature based on CCC that has a powerful ability to predict prognosis and immunotherapy response in patients with HNSCC.
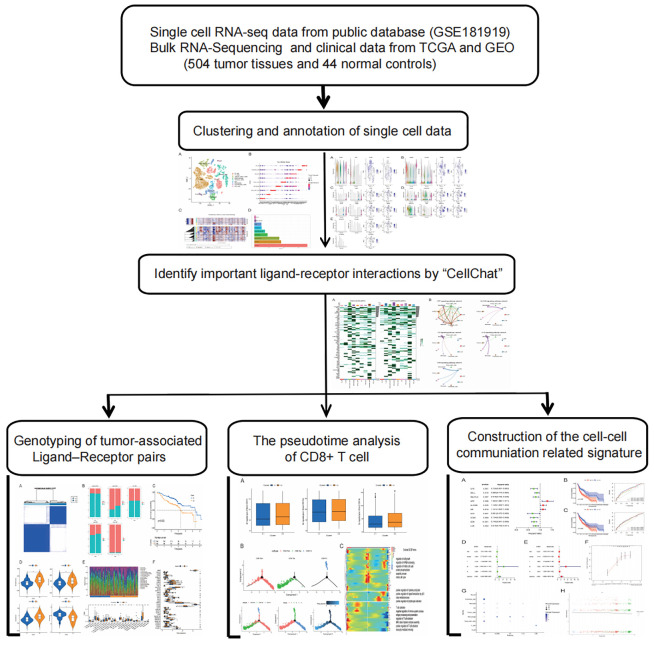

## Introduction

Head and neck squamous cell carcinoma (HNSCC) ranks sixth among the world’s most common carcinomas, and 2018 alone witnessed 890,000 new cases and 450,000 deaths (1). The worldwide incidence and prevalence of HNSCC is on the increase, with the incidence rate presumably up 30% by 2030 (a yearly increase of 1.08 million) (GLOBOCAN) (2). Men have a 2-4 times greater risk of HNSCC than women. The mucosal epithelium lining the mouth, pharynx, larynx, and sinuses is the origin site of HNSCC (3). The histological progression model of HNSCC indicates that dysplasia follows mucosal epithelial hyperplasia and invasive carcinoma follows carcinoma in-situ (4). Specific genetic events have been found to be enriched at each stage of progression and have been identified. Notably, unlike most cancers, where tumorigenesis is generally driven by cancerogenic mutations, the onset of HNSCC tends to be associated with the inactivation of cancer suppressor gene, as in the case of early-stage CDKN2A and TP53 (encoding p16INK4A and p53, respectively) and later-stage PTEN (encoding phosphatase and tensin homologous) (5–7). High-throughput sequencing analysis of tumor tissues is based on the analysis of mixed samples of millions of cells, reflecting the overall genomic characteristics of cells, but ignoring the heterogeneous interference of tumor microenvironment, resulting in insufficient accuracy. Thanks to the development of single-cell sequencing technology, the accuracy of detecting gene variation in tumor cells has been improved, providing a new idea for revealing the occurrence and development of HNSCC. It can be used to compare the differences of single cell genome, transcriptome and epigenetic group in tumor primary site, metastatic site, metastatic lymph node and peripheral blood (8).

This extensive association between tumor cells and immune cells is often closely related to patient outcomes. Cell communication is usually mediated by ligand-receptor interactions, and the association of tumor cells with other cells is prevalent in HNSCC tumor microenvironments (9). For example, loss of PD-L1 (programmed cell death 1 ligand 1), PDCD 1 (programmed cell death 1), CD38 molecule-adenosine receptor, and HLA-associated ligand-receptor interactions can inhibit CD8+ T cells’ tumor-killing function, respectively (10–12). In cellular communication between cancerous cells and CD8+ T cells, certain ligands or receptors are closely related to cellular state; Blocking the inhibitory programmed cell death 1 (PD-1) pathway has been shown to restore immune function (13). Apart from that, macrophages can be polarized in different directions under different microenvironments and intercellular interactions. According to the different activation state, function and secretion of cytokines, macrophages are classified into pro-inflammatory and anti-inflammatory, which activated M1 and M2 type macrophages, respectively (14).

Macrophages, either of M1 or M2 type, are closely associated with inflammatory response. When macrophages are recruited into the inflammatory tissue, they further release more pro-inflammatory cytokines, which include IL-6, TNF, IFN-γ, EGF, various proteases and reactive oxygen species (ROS). A large amount of evidence shows that inflammatory responses at tumor sites can promote tumor growth and progression. Inflammation and immune evasion are thought to be hallmarks of cancer (15). If the tumor suppressor genes of normal cells are mutated, chronic inflammatory tissues are prone to become cancerous and produce tumors in the long run. But macrophages have a new identity as tumor-associated macrophage (TAM) after carcinogenesis. In the beginning, TAM was believed to be an anti-tumor effector cell (M1-like macrophages), which could kill tumor cells or present tumor antigens to induce the body’s immune response and eliminate tumors. However, with the deepening of research, people found that the main role of TAM (M2-like macrophages) was to promote tumor and suppress immune. The clinical employment of immunotherapy targeting immune checkpoints has substantially enhanced clinical effect and altered the therapeutic paradigm for HNSCC. However, the clinical benefit of immunotherapy remains limited to a subset of patients with HNSCC.

It is therefore one of the most pressing problems to develop the optimal predictive models and explore new biomarkers for prognostic and therapeutic prediction through screening of cell-cell communication genes in HNSCC.

## Materials and methods

### Data collection of HNSCC samples

In this study, 20 HNSCC samples (GSE181919) with RNA-seq data were included from public database. 508 TCGA samples with clinical data and expression profiles (44 in the normal group and 504 in the tumor group) were used as the experimental group. 270 HNSCC samples (GSE65858) from a separate GEO cohort were used for the test group. (https://www.ncbi.nlm.nih.gov/geo/). We analyzed the RNA-seq data through the “Seurat” package of R software (version 4.2.1) (16). For data quality control, we applied three filtering measures to the original matrix of the cell: genes that found expression in over three single cells, cells that express 50 plus genes, and cells with less than 5% mitochondrial genes. We first conducted data quality control, normalized and calibrated single-cell data by the functions “scaledata” and “normalizedata”. Then, we apply the “RunPCA” function to the principal component analysis (PCA) and select 1:15 according to “ElbowPlot” (17). As principal component, “FindVariableFeatures” were used to find 2000 high-variable genes, and “FindNeighbors” and “FindClusters” were used for cell clustering analysis. Then, random neighborhood embedding (t-SNE) of T-distributions is performed using “RunTSNE” function visualization. Using Wilcoxon the Mann - Whitney test calculation of each cluster differentially expressed genes (DEGs). For the identification of marker genes within the cluster, we employed cutoff thresholds (adjusted P-values < 0.05 and avg_logFC > 1). For clustering annotation, we use “singleR” reference data from HumanPrimaryCellAtlasData annotation of cells carried out based on the reference (18). T cell data were extracted from cell population, data quality control and calibration were performed again, and T cell population was performed again with reference to T cell gene annotation (19). At the same time, the first 1000 significant genes were used as input of “Monocle2” algorithm for subsequent sequencing to construct developmental tracks of different CD8+ T cell subsets (20).

### Identify important ligand-receptor interactions

The R packet (CellChat) contains a database of human ligand-receptor interactions that enables the analysis of intercellular communication networks from annotated ScRNA-seq data for different cell clusters (21). First, we used “CellChatDB.human” to evaluate the major signal inputs and outputs of cell populations in all HNSCC samples. We then use the heatmap function to summarize all the signal paths to see which one contributes the most, After the results of cell interactions at the signaling pathway level were obtained, univariate Cox regression analysis was utilized for prognostic evaluation of ccc in TCGA HNSCC patients with overall survival(OS), and to determine p < 0.05 genes was the prognostic related ccc genes.

### Tumor associated ligand-receptor genotyping

According to the expression profiles of genes related to cell-to-cell communication, different communication characteristics were determined by cluster analysis, and HNSCC patients were classified for further evaluation. Kaplan-Meier(K-M) survival analysis, clinical characteristics analysis, immune microenvironment analysis, immune cell infiltration analysis and CD8+T cell differentiation correlation analysis were performed. Thus, the interaction between immune cells and tumors was elucidated. We increased the clustering of the variable (k) from 2 to 9 and repeated this 1000 times using the “conesusclusterplus” package (22) to ensure optimal classification stability. Finally, the consensus clustering matrix (k = 2) was used to divide the HNSCC patients into two different genomic subtypes: gene cluster 1 (C1) and gene cluster 2 (C2). A Log-rank test and K-M survival analysis were performed to show the OS differences. Surviving receiver operating characteristic (ROC) curves (1, 3, and 5 years, respectively) and their area evaluation curves (AUCs) were compared with other clinical features with a view to evaluating the prognostic effect of the two groups of cell communication molecular subsets. The “Monocle2” R package was used for T cell locus and pseudotime analysis (23). Pseudotime analysis was based on the sequencing of the first 1000 significantly different genes of CD8+T cell types, showing the metastasis locus of different cell clusters. At the same time, the branch differential genes were preserved, and the proportion of the locus branch differential genes in the molecular subtype was calculated. On basis of T cell locus different branches differential genes, Gene Ontology (GO) analyses was made with a modified P < 0.05 (24). We applied “ESTIMATEScore” to calculate immune markers, matrix markers and estimation markers for each sample (25). “CIBERSORT” was used to classify immune-related cells, and the immunoinfiltrate status was analyzed according to GEO data set, which was shown in box chart form (26). Wilcoxon symbolic rank test was conducted for comparison between groups in terms of infiltrating immune cell, immune function and immune checkpoints.

### Establishment of a prognostic signature based on cell-cell communication genes

Single-factor Cox analysis was carried out, in which R packet “Survival” was employed to screen the intercellular communication genes with independent prognosis. The optimal prognosis model was then determined and developed on the basis of the Multivariate Cox regression analysis and the “glmnet” R package (27). Risk markers of HNSCC samples were calculated according to relevant regression parameters and gene expression levels: $\mathrm{Risk score}=\sum_{i=0}^{n}coefficient$ x expression of cell-cell communication genes^i^.

### Verification of the prognostic signature for HNSCC

The receiver operating characteristic (ROC) curve of 1-, 3-, and 5-years was established for effectiveness assessment of the cell-cell communication genes signature (cccgs) model. K-M survival curves were employed to compare the risk groups in total survival diversities. The univariate and multivariate Cox regression analysis was performed for cccgs risk score assessment as a hidden independent prognostic predictor of HNSCC. A nomogram was constructed to predict survival rate in patients by incorporating such factors as age, gender, stage and risk score. The calibration curves were utilized to assess the predictive power of the nomogram.

### Statistical analysis

Statistical analysis was made with the aid of R language (version 4.2.1). The Chi-square test and Wilcoxon signed-rank tests were performed to constant variable data. Pearson correlation analysis was utilized to assess the association between variables. P < 0.05 was considered statistically significant.

## Results

### Infer the status of tumor cells and T cells

**Figure 1:**
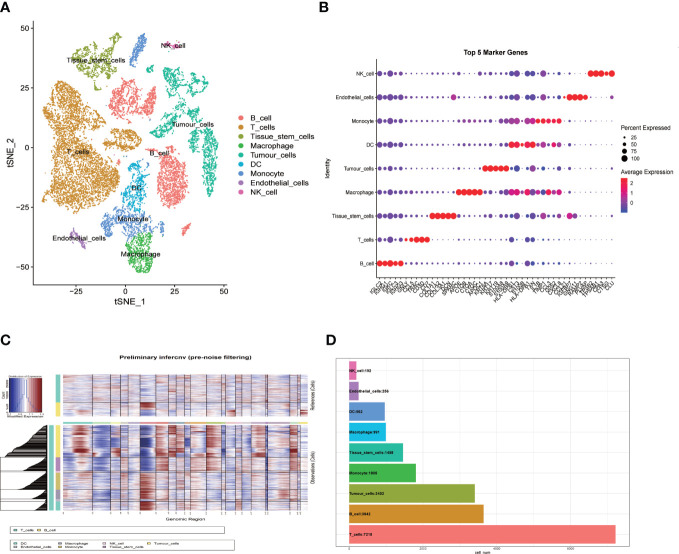
Single-cell RNA-sequencing analysis identifies cell marker genes. **(A)** t-SNE plot of 23088 cells colored by various cell clusters from 20 primary HNSCC samples; **(B)** bubble diagram showing the top 5 marker genes in each cell cluster; **(C)** The hierarchical heatmap showing large-scale Copy-number variation analysis(CNVs) in tumor lesions; **(D)** Statistics of cell numbers in each cell cluster.

With single cell RNA-seq data of GSE181919 as the basis, gene expression profiles of 23088 cells were obtained from 20 primary HNSCC samples (**Figure 1A**). PCA was performed to the top 2000 variable genes for dimensionality reduction, and the identification of nine cell clusters was completed (**Figure 1B**) and annotated with reference to a dataset from the Human Primary Cell Atlas. T cells and B cells serve as normal reference cells, using the “inferCNV” R package, we found that these epithelial cells had significant chromosomal copy number changes and labeled them as tumor cells (**Figure 1C**). Through the “singleR” package, we labeled the cell types after clustering, and the top 5 genes of each cell type were illustrated in **Figure 1B**. The number of cells contained in each cell type was illustrated in **Figure 1D**. We found that T cells were the most abundant (7218 cells), but fewer NK cells and endothelial cells.

**Figure 2:**
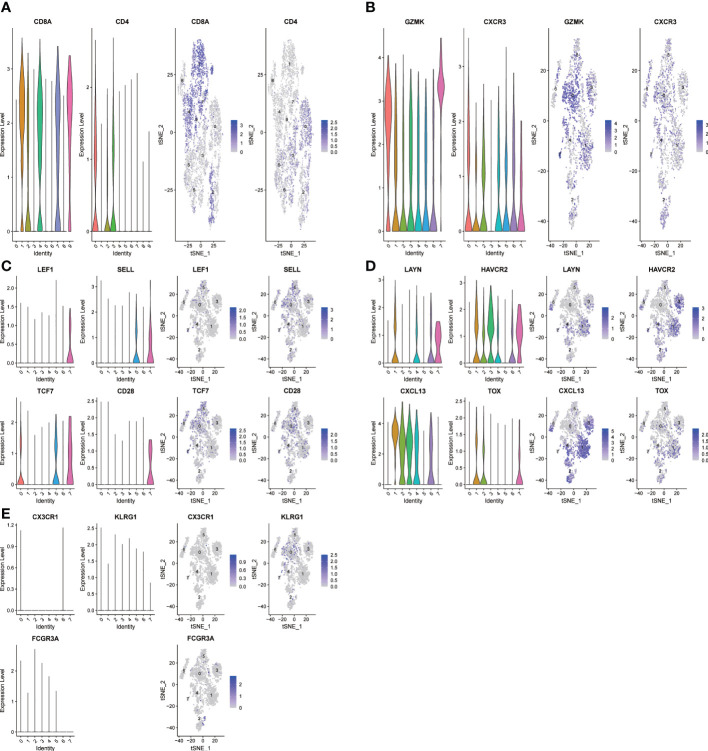
The t-SNE projection of T cells from HNSCC patients and the functional description of each cluster is determined by the gene expression characteristics of each cluster. **(A)** Cluster 1,4,7,9-CD8A:CD8+ T cells;Cluster 0,3-CD4:CD4+ T cells; **(B)** Cluster 0-CD8-CXCR3: effector memory CD8+ T; **(C)** Cluster 7-CD8-LEF1: naive CD8+ T cells; **(D)** Cluster 1-CD8-LAYN: exhausted CD8+ T cells; **(E)** Cluster NULL-CD8-CX3CR1: CD8+T effector cells.

The expression profiles of 7218 T cells were again clustered and divided into 9 subgroups. CD8+T cells and CD4+T cells were distinguished according to CD8A and CD4 marker genes (**Figure 2A**). In cluster 1, 4, 7 and 9 were CD8+T cells, cluster 0 and 3 were CD4+T cells. Since T cell subsets can still be finely divided, we selected several classical markers of functional status and the expression levels of the cluster differential genes indicated cell status of CD8+ T cells. The marker genes of T cells came from a single-cell study conducted by Zemin Zhang’s team (19). In the paper, published in Nature in 2018, Zemin Zhang’s group screened CD4 or CD8 T cells by flow cytometry. the T-cell subsets and their highly expressed genes at different stages of differentiation are shown in their paper (extended data **Figure 3**). So we directly selected their organized marker genes to identify the different stages of T cell differentiation. Specific marker genes are included in the **Table 1**. For example, the marker genes of CD8+T naive (CD8+Tn) are LEF1, SELL,TCF7 and CD28, and the marker genes of CD8+T effector (CD8+T eff) are CX3CR1,KLRG1 and FCGR3A. CD8+T Effector memory (CD8+ Tem) marker genes were GZMK and CXCR3, etc., and CD8+T exhausted (CD8+Tex) marker genes were LAYN,HAVCR2,CXCL13 and TOX, etc (**Figures 2B–E**). As shown in **Figure 2E**, we did not find effector T cells (CD8+Teff) in the tumor sample group with direct killing function to the tumor, which is attributed to long-term interplay between cancerous cells and the microenvironment.

**Figure 3:**
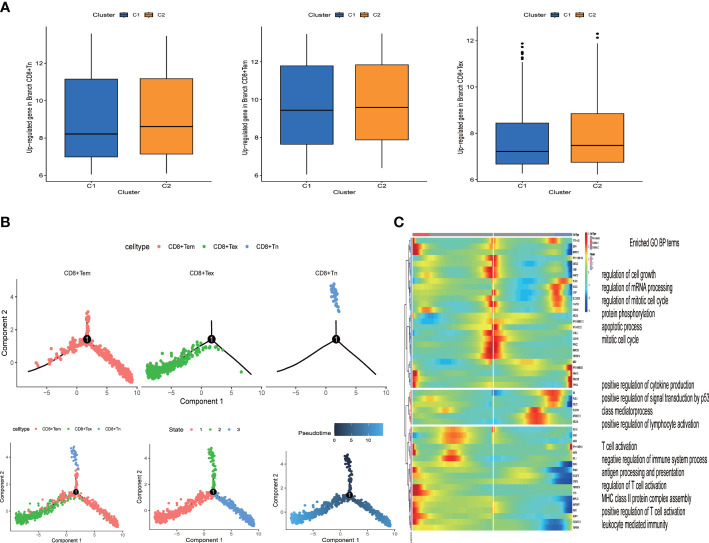
Identification of heterogeneous genes associated with differentiation trajectories of CD8+ T cells. **(A)** Different T cell states in two clusters. **(B)** Differentiation trajectory analysis of CD8+ T cells. **(C)** The heat map revealed that CD8+ T cells could exhibit three expression patterns after differentiation; GO enrichment analysis of heterogeneous genes associated with CD8+ T cells differentiation.

**Table 1:** CD8+ T cell markers at different stages.

T cell cluster names	Representative genes	Functional properties
CD8-LEF1	CCR7,LEF1,SELL,TCF7,CD27,CD28,etc	CD8^+^ T naive
CD8-GZMK	GZMK,CXCR4,CXCR3,CD44	CD8^+^ T effector memory
CD8-LAYN	HAVCR2,CXCL13,PDCD1,LAYN,TOX,IFNG,etc	CD8^+^ T exhausted
CD8-CX3CR1	KLRG1,CX3CR1,FCGR3A,FGFBP2,PRF1,GZMH,etc	CD8^+^ T effector

### The landscape of cell-cell communication Characterized by ligand–receptor interactions

**Figure 4:**
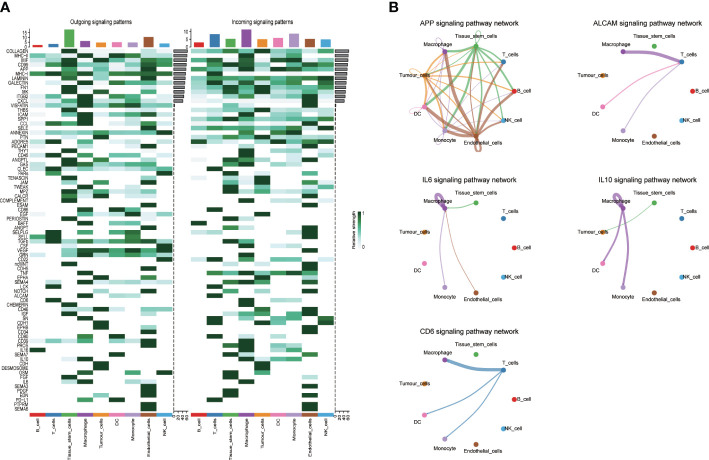
Interaction plot of tumor cells and intercellular communication networks. **(A)** Heatmaps of the overall (comprising both outgoing and incoming) signaling flows of each cell population mediated by individual signaling axes in control and HNSCC sample; **(B)** The circle plot showed the inferred intercellular communication network for five model related signaling pathways. The thicker the line represented, the more the number of interactions, and the stronger the interaction weights/strength between the two cell types.

The “Cellchart” software package was used to explore the signaling pathway network and differential ligand-receptor pairs between HNSCC and other cells. We selected the known ligand-receptor pairs in the “CellChatDB.human” database to identify the overexpressed genes and the probability of expression interaction in the single cell dataset, and obtained the global cell interaction network and significant pathways. As shown in the **Figure 4**, the darker the intercellular signaling pathway, the stronger the correlation.

### Genotyping of tumor-associated ligand–receptor pairs

**Figure 5:**
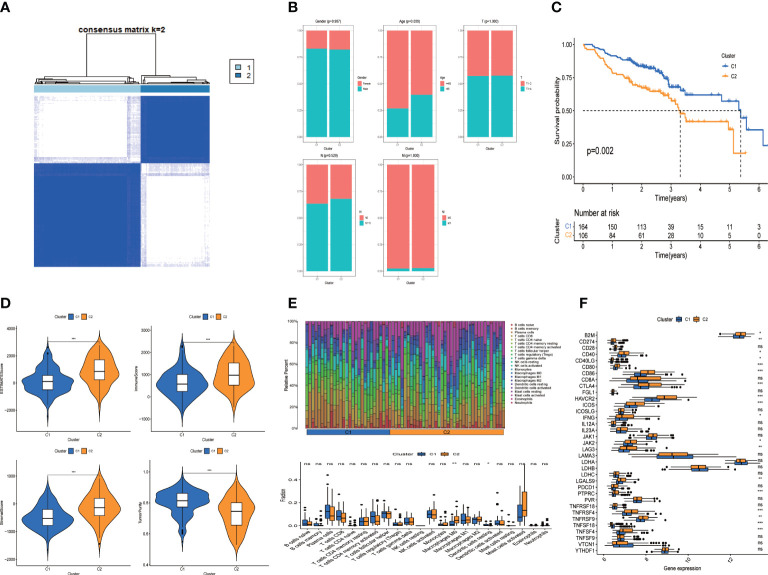
Clusters analysis. **(A)** Tumor related cell-cell comunication genes divided into two clusters; **(B)** Comparison of clinicopathological features in clusters **(C)** K-M survival curves of OS in clusters; **(D)** Immune-related scores in clusters; **(E)** Heatmap and Box-type drawing of immune cells in clusters; **(F)** Different expression of checkpoints in clusters. Adjusted p-values were showed as: ns, not significant; *p< 0.05; **p< 0.01; ***p< 0.001.

R package “cellchat” was used to obtain cell communication data, and consistent clustering of tumor-related communication genes was performed, with the best effect when clustering into 2 categories (**Figure 5A**, K=2). C1 subgroup includes 164 HNSCC samples, while C2 subgroup includes 106 samples. K-M analysis showed poor prognosis in the C2 subgroup (**Figure 5C**, P=0.002). Then, the association between the clinicopathological elements and Genotyping showed that the Gender, T stage, N stage and M stage (P > 0.05) were not greatly different between C1 and C2 (**Figure 5B**), but there were more patients over 60 years old in C2 group (P < 0.05).

### Immune-related characteristics and immuno-therapy forecast of genotyping groups

We further assessed the difference of tumor immune microenvironment of C1/C2 subgroups. Patients with HNSCC in C2 group scored higher than those in C1 group in terms of estimate score, immune and stromal marks (**Figure 5D**). Correspondingly, the tumor purity was lower in the C2 group. We examined the condition of immune cell infiltration in each group and concluded that B cells, plasma cells, most T cells, activated NK cells and dendritic cells were upregulated in C1 subgroup, while M0 and M1 Macrophages were infiltrated more in C2 group (**Figure 5E**).

To investigate the immunotherapeutic effect, we analyzed the relations between genotyping Groups and Immune checkpoint genes. The result revealed that immune checkpoints in C2 group were considerably higher than those in C1 group (**Figure 5F**, P < 0.05), suggesting that tumor cells may use immune checkpoint molecules to suppress the function of immune cells and form immune escape. That explains why patients in C2 has a worse prognosis than those in C1 group (**Figure 5C**).

### The tumor-associated genotyping state and the CD8+ T exhausted state are synergistically associated with clinical benefit

From the expression data of single cell sequencing, we identified three states of CD8+T cells (naive, effector memery and exhausted state). We found that CD8+Tex state was highly expressed in the C2 group, while CD8+Tn and CD8+Tem were no different in the two groups (**Figure 3A**). The overall survival rate of the C2 group was lower (**Figure 5C**). Due to the direct killing effect of T cells on the tumor, the overall survival rate of patients in the group with more CD8+Tex cells was lower, indicating that the depletion of CD8+ T effector was consistent with the poor clinical prognosis of HNSCC patients. In order to further understand the evolution process of CD8+T cells from naive to exhausted in tumor microenvironment, we predicted the genetic changes of T cells by pseudo-temporal analysis (monocle2), and analyzed the important role of T cell states by Go analysis (**Figure 3C**). We used the R package “monocle2” to construct the intercellular change locus to reconstruct the change process of cells over time, and analyzed the transcriptional gene changes experienced by navie T cells when they moved from the initial state to the state of T cell exhausted, as shown in **Figure 3B**. In pseudotime series analysis, the three main T cell types were respectively CD8+Tn, CD8+ Tem and CD8+Tex. The starting point of state1 was considered to be CD8+Tn, and some T cells transformed into state 2 (CD8+Tex) over time. The other part was transformed into CD8+ Tem cells (**Figure 3B**). The heat map of T cells’ transcriptional gene changes from node one to branch2 and branch3 was shown in **Figure 3C**. After obtaining the differential genes of each branch, the functional enrichment, GO analysis included, indicated that the CD8+T cell marker genes were mostly associated with immune features, such as T cell action, positive regulation of cytokine and lympocyte production.

### Establishment and verification of the five-gene prognostic signature based on cell-cell communication genes

**Figure 6:**
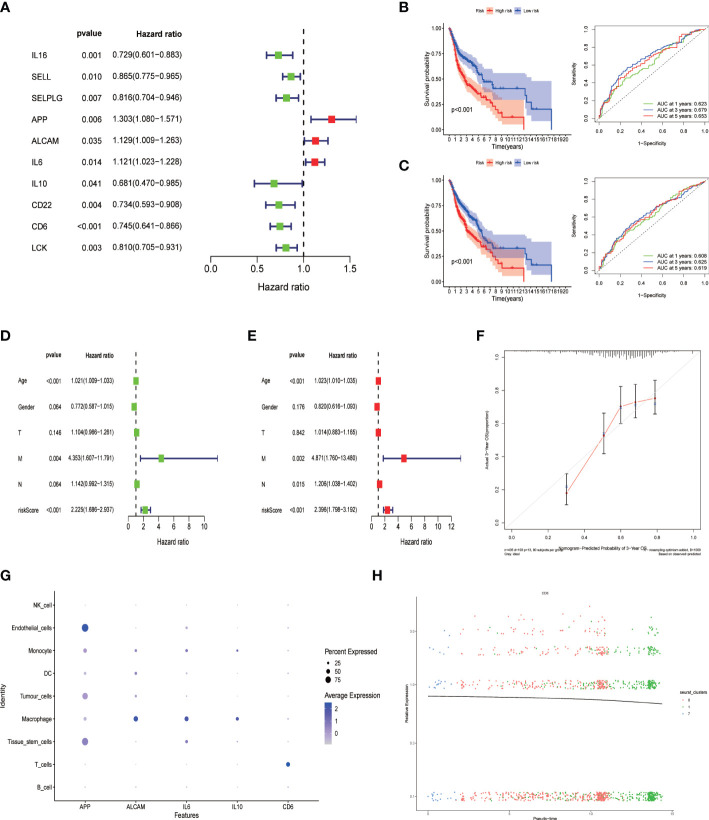
Construction of cell-cell communication-related prognostic signature. **(A)** Univariate Cox regression analysis of prognostic genes associated with cell-cell communication. Then a 5-gene prognostic model(APP, ALCAM, IL6, IL-10 and CD6) was constructed by Multifactorial stepwise regression; **(B)** Kaplan-Meier prognostic analysis and Time-dependent ROC curves of signatures in the training dataset. **(C)** Kaplan-Meier prognostic analysis and Time-dependent ROC curves of signatures in the testing dataset. **(D)** Univariate and **(E)** multivariate Cox analysis of risk score and clinical factors. **(F)** Calibration curves plot; **(G)** The expression level of five prognostic gene signature in different Cell population; **(H)** CD6 gene expression changes with CD8+ T cell differentiation.

Univariate Cox regression analysis was performed for prognostic evaluation of cell communication-related genes in HNSCC patients, genes with p < 0.05 were considered to be prognostic genes (**Figure 6A**). Finally, the prognostic model was constructed by 5 genes (APP, ALCAM,IL6,IL10,CD6) using multivariate Cox regression analysis. To verify the prognostic ability of the model gene, we calculated the area under the curve (AUC) using the software package “timeROC”. Survival analysis was conducted under the Kaplan-Meier method and R package “survminer”. And the log-rank test was performed for comparing survival distributions of the groups. In addition, the predictive power of the signature was validated using GEO independent data sets, survival analysis and the AUC. The area under the ROC plot in train group was 0.623, 0.679 and 0.653 respectively for one year, three years and five years. Kaplan-Meier survival analysis showed that the low risk group had a higher survival probability than the high risk group (**Figure 6B**, p < 0.001). The outcomes were confirmed by GSE65858 datasets as validation cohorts (**Figure 6C**, p < 0.001). Univariate and multivariate Cox regression analyses suggested that the risk mark was an independent prognostic factor of total survival in patients with HNSCC (**Figure 6D**; HR = 2.225, 95% CI: 1.686–2.937, P < 0.001, **Figure 6E**; HR = 2.396, 95% CI: 1.798–3.192, P < 0.001). In accordance with the calibration cures, the risk model had great predictive power through comparison with the ideal model (**Figure 6F**). Model-related genes were mainly expressed in macrophages and T cells (**Figure 6G**). The expression of CD6 gene decreased with T cell depletion (**Figure 6H**).

## Discussion

With the advancement of single cell sequencing technology, researchers are exploring more and more cell-cell communication patterns in HNSCC samples to analyze the molecular properties of cancerous cells and immune cells in TME.

It is not difficult to see that tumor cells, as transmitters, are characterized by enhancing the close connection between each other, promoting proliferation, and evading immune surveillance by the landscape of cell-cell communication characterized by ligand–receptor interactions. Such as macrophage migration inhibitor (MIF), polytrophic factor (PTN) and cell adhesion related genes (cadherin, cadherin-1 and desmosomes) are highly expressed in the signaling pattern of tumor cells. Macrophage migration inhibitors (MIF) are not only pro-inflammatory mediators, but also promote the occurrence of tumors. The expression of MIF had positive correlation with angiogenic growth factor expression, microvascular density, and tumor-related new blood vessel formation. It has been shown to promote tumor hypoxic adaptation by stabilizing hypoxia-induced HIF-1alpha (28). The PTN gene is highly expressed in cancer cells. It is a secretory growth factor that can cause tumor cells to proliferate and migrate and promote the formation of tumor blood vessels, self-renewal and stem cell procedures (29). Inhibition of the expression of this gene can effectively control the excessive proliferation of tumor cells, thus achieving the purpose of inhibiting the development of tumor. In addition, we found that CDH1, CDH, and DESMOSOME ligand receptor pairs have strong associations inside tumor cells. CDH and CDH1 genes are associated with cellular adhesion alongside calcium ion binding. CDH gene family plays a mediating role in intercellular adhesion according to the findings by akeichi et al. Despite the fact that CDH gene adhesion molecules are important for cellular selective aggregation, they have close relations with the invasive and metastatic behavior of tumor cells in cancer progression (30) Desmosomes have intermediate filaments in the cells underneath that help anchor the junction. Such tight connections between tumor cells may also be associated with drug resistance in solid tumor clumps, such as elevated desmosomal core glycoprotein-2 levels in pancreatic cancer cell spheres, which also lead to increased resistance to doxorubicin (31). In addition, High expression of CD46 related genes in tumor cells. CD46, as a cofactor of complement factor I and a co-stimulatory factor of T cells, induces CD4+ differentiation into T regulatory 1 cells. T-regulatory 1 cells suppress immune responses by secreting interleukin-10. This is thought to protect against autoimmunity, leading to tumors being unrecognised by the immune system (32).

We also find that antigen presentation (MHC-1), cell adhesion molecules involved in T cell migration (ADGRE5, SELPLG, SELL), and ligands-receptors associated with activation of inflammatory responses and secretion of pro-inflammatory cytokines (LCK, CD6) were expressed in T cells, indicating the killer potential of T cells against tumor cells (33–35). The lack of an antigen processing mechanism (MHC-I and MHC-II) mediated interferon-gamma response receptor in tumor cell receptors may result in their failure to be recognized by immune cells (36).

Immune cell infiltration in TME is of vital importance to tumor progression and affects prognostic effect remarkably. We then adopted estimation and cell classification algorithms to compare the ccc subtypes in terms of immunocyte infiltration abundance. The abundance of immune cell infiltration has a close relationship with the prognosis of HNSCC patients, especially T cells transit from a naive cell state to a depleted state. The depletion state of CD8+ T correlated with the survival rate of HNSCC patients, and the higher the T consumption, the lower the survival rate of patients. Based on the T cell pseudotime sequencing analysis heat map, we found that mature T cells that have never been stimulated by antigens express mainly the genes of cell cycle, cell growth and differentiation, and mitosis. The initial T cells (CD8+Tn) and memory T cell (CD8+ Tem) were stimulated by pMHC presented by DC and activated to differentiate into effector T cell (CD8+ Teff) (37). In our study, no significant marker genes of effector T cells were found in the HNSCC samples included. This may be due to the depletion of T cells under the long-term suffering of tumor, but there are still memory T cells and the main functions of T cells are preserved, such as T cell activation and regulation, antigen processing and presentation, leukocyte mediated immunity, in contrast, exhausted T cell (CD8+Tex) showed downregulation and silencing of most of immunofunctional genes. The more T cell depletion states, the more significantly correlated with poor prognosis in HNSCC patients. Our study shows that T cell status acts a central part in immunization from carcinoma.

In addition, the higher the content of tumor-associated macrophages (TAM) in the tumor, the lower the survival rate of patients, which means that they are not anti-tumor effector cells, but display the side of promoting tumor and immunosuppression. TAM are the main components of tumor invasion immune cells in the tumor microenvironment and plays a mediating role in inflammation related to tumor progression. TAM promotes the development and metastasis of tumor, stimulates angiogenesis, mediates immunosuppression, and limits antineoplastic activity of chemoradiotherapy. There is evidence that increased TAM infiltration and overexpression of genes associated with macrophages is related to poor prognosis in the majority of solid cancers (38). We also found that patients detected of high immune checkpoint expression had poor survival expectation, indicating that immune checkpoint blockade method is applicable to patients with HNSCC.

In order to further screen the key role of ccc in patients’ prognosis, a new prognostic feature based on ccc was developed in the TCGA database for HNSCC patients and was well validated in a GEO data set. The model was composed of five prognostic genes, namely APP, ALCAM, IL6, IL10 and CD6. There is no point that ALCAM, IL6, and IL10 had high expression in TAM and CD6 had high expression in T cells. Over expression of ALCAM (activated leukocyte adhesion molecule) had direct relations with increased TAM cell aggregation. ALCAM, also known as CD166, is a cell adhesion protein present in a variety of cell types, mediating the adhesion of homologous and heterologous cells *in vivo*. CD6 is a recognized binding partner of ALCAM, promoting T-cell activation and proliferation *via* its interactions with CD6 (39). Nevertheless, a study of 105 cases of oscc showed a significant reduction in overall survival in patients with negative ALCAM cytoplasmic and E-cadherin membrane staining. Similarly, groups of patients with cytoplasmic/nucleus beta-catenin and cytoplasmic ALCAM staining were also associated with lymph node metastasis and advanced cancer (40). Some other relevant findings show that 47% of OSCC tumors are tested positive for ALCAM staining and that this is associated with short-term survival in patients and appears to be an independent factor (41). This may be related to the acquisition of an aggressive phenotype and the metastatic spread of cancer cells. Due to continuous antigen exposure (tumor), exhausted T cells gradually lose their effector function (CD8+ Teff), and CD6 is also down-regulated, decreasing its killing effect on tumor. Those result indicate that high ALCAM and low CD6 expression indicates poor prognostic effect, a conclusion in consistency with our previous findings.

In addition, IL6 and IL10 are a major immunomodulatory cytokine that acts on many cells of the immune system. Study shows that colorectal cancer (CRC) conditioned macrophages regulated epithelial–mesenchymal transition program to promote the invasive and migrating behavior of CRC cells by secreting IL6 (42). On the contrary, IL10 has far-reaching anti-inflammatory functions and can limit the excessive tissue destruction caused by inflammation (43). This explains IL-10 as a protective factor in our model. Those results suggest that eliminating the molecular features exhibited by tumor cells in combination with immunotherapy promises to be a novel therapy.

There are also some deficiencies with the study. Because of the absence of sequencing data from cells undergoing physical interaction, we analyzed the association between CCC and cell state, which could improve reliability. Currently, there appear some methods using spatial transcriptome data, and ccc integration that will rely on physical contact and chemical signals will be a future trend. This study is founded utterly on public database and part of the crucial genes or outcomes needs external verification through further experiments, including immunohistochemistry to show the role of key genes. In addition, it is difficult to answer whether there are dynamic changes in CCC in this study. The effect of tumor and immune cell infiltration on the patient cannot be directly representative of the part played by CCC. Yet, the isolation of CCC from different cell subsets remains a challenge.

## Conclusions

In a nutshell, a ccc-based five-gene signature was identified and confirmed to have strong predictive power for prognostic response in patients with HNSCC. cccgs may affect the molecular characteristics and immune microenvironment of HNSCC development. Our results provide insights for predicting outcomes and identifying therapeutic targets for patients with HNSCC.

## Data availability statement

The datasets presented in this study can be found in online repositories. The names of the repository/repositories and accession number(s) can be found in the article/supplementary material.

## Ethics statement

Ethical review and approval was not required for the study on human participants in accordance with the local legislation and institutional requirements. Written informed consent for participation was not required for this study in accordance with the national legislation and the institutional requirements.

## Author contributions

JW designed the study and carried out the data analyses. H-CS, CC, J-DH, JQ and TJ interpreted the results. W-BJ and SZ draft the manuscript. X-WQ and H-LW revised the manuscript. All authors read and approved the final manuscript. All authors contributed to the article and approved the submitted version.
